# ABO blood group, ABO mismatched transfusions and leukoreduction of transfusions in hemostatic resuscitation studies

**DOI:** 10.3389/fbioe.2024.1437471

**Published:** 2024-09-16

**Authors:** Phuong-Lan Nguyen, Akua Asante, Majed Refaai, Neil Blumberg

**Affiliations:** Transfusion Medicine Division, Department of Pathology and Laboratory Medicine, University of Rochester Medical Center, Rochester, NY, United States

**Keywords:** transfusion, ABO, whole blood, platelets, plasma

## Introduction

Biocompatibility and immunologic compatibility are important contributors to transfusion efficacy and safety in trauma patients. Some studies suggest that low-titer group O whole blood may be superior to conventional current use of component therapy—separate units of red cells, plasma, platelets and cryoprecipitate (Yazer et al., 2022; Belin et al., 2017). This superiority has been attributed to more physiologic composition and logistic advantages. What has not been considered is the ABO blood group of the patient, or whether ABO incompatible antibody and soluble or cellular antigens were infused, and in what quantity. This is important as ABO non-identical transfusions have been associated in observational studies and *in vitro* modeling with increased bleeding (Magid-Bernstein et al., 2021; Blumberg et al., 2001), impaired platelet function and thrombin generation (Refaai et al., 2013), damaged endothelial cells (McRae et al., 2021), and increased mortality (Blumberg et al., 2001) compared with ABO identical transfusions.

Similarly, whether the whole blood or components have been leukoreduced has not been considered in most analyses. This is important, as leukoreduction of transfusions has been shown to reduce post-operative infection and mortality in randomized trials in surgical patients (Blumberg et al., 2007; Simancas-Racines et al., 2019; Fergusson et al., 2004).

## ABO type of recipients and ABO identical transfusions

Traditional concepts in the field of hematology/immunology are that group O red cells, group AB plasma and any ABO group platelets and cryoprecipitate are equally effective and safe as ABO identical. Recent data from many clinical settings suggest that these concepts are outdated. Infusing even smaller amounts of anti-A and anti-B (e.g., group O red cells) on rare occasions leads to severe hemolysis (Barjas-Castro et al., 2003). 

Infusing incompatible soluble A and B antigen to patients with the corresponding antibody is associated with increased bleeding, vascular injury, and impaired hemostasis in a variety of clinical settings (Blumberg et al., 2024). It will be important in studies comparing various whole blood or component therapy regimens to document the receipt of minor and major incompatible ABO transfusions, and assess the quantity of incompatible antigen or antibody received. Given that methods of defining “low titer” differ, this variable should be accounted for, given that more antibody is probably harmful. Comparisons with current methods of component therapy are not reassuring, since this involves infusing ABO incompatible antigen and antibody to almost all recipients.

With evidence that use of group A plasma or low titer group O whole blood is equivalent or perhaps safer than traditional concepts of ABO, the paradigm needs to shift. One reason why low titer group O whole blood might be better is that it is ABO identical with 45% of recipients, and involves the infusion of smaller amounts of incompatible antibody. 

ABO incompatible antigen and antibody content is of potential importance. Current data suggest that moving towards resuscitation with blood containing minimal ABO incompatible antigen and antibody might improve efficacy and safety of resuscitation from life threatening hemorrhage.

## Patient ABO type and outcomes of hemostatic resuscitation

Patients who are group O bleed more (Ozbay et al., 2016). Patients who are group A, B and AB bleed less, but have a greater tendency toward thrombosis (Masseli et al., 2024). This has been attributed to striking variances in von Willebrand factor levels, with AB the highest and group O the lowest. Whatever the cause(s), it would be worthwhile to document the survival rates early on and at discharge of patients of each ABO type. One would expect earlier mortality due to bleeding in group O patients. Group B and AB patients are most likely to receive ABO mismatched transfusions during resuscitation, and this might, speculatively, increase later deaths due to organ injury, sepsis, etc. Anti-A is of highest potency, and group A patients have highest density of A antigen, thus group A patients might be particularly vulnerable to hemolysis and organ injury.

## Leukoreduction of whole blood

There is a body of opinion that is skeptical of the benefits of leukoreduction of whole blood for trauma resuscitation. A major reason is that the one randomized trial showed no benefit of leukoreduction in this clinical setting (Nathens et al., 2006). However, that study, like most such studies, did not account for receipt of ABO incompatible antigen and antibody, which likely influence morbidity and mortality. In addition, a number of meta-analyses of leukoreduction in surgery claimed that there was no convincing benefit (Kunz and Guyatt, 2006; Vamvakas, 2002). However, this group’s multiple meta-analyses employed data that included up to 35% of patients who received no transfusions whatever. This is misleading since non-transfused patients cannot benefit from leukoreduction. Including them in the analysis through a well-intentioned but methodologically unsound attempt to create an “intention to treat” analysis favors the null hypothesis. Thus these studies involve experiments that were not actually performed, and have misled the medical profession for decades.

Subsequent meta-analyses have avoided such misleading practices and demonstrated striking benefit to leukoreduction of transfusions for surgical patients. This is reductions in post-operative infections by about 30%–40% and reductions in mortality in cardiac surgery (Blumberg et al., 2007; Simancas-Racines et al., 2019; Fergusson et al., 2004). Leukoreduction minimizes the infusion of allogeneic white cells, free DNA, neutrophil extracellular traps and histones, which are associated with organ injury and mortality in animal experimental models (Thomas et al., 2012). Implementation of leukoreduction in our hospital led to an 80% reduction in reports of transfusion-related lung injury and 50% reduction in reports of congestive heart failure (Blumberg et al., 2010). Leukoreduction in our setting (an 800+ bed academic medical center) was associated with striking reductions in cardio-respiratory morbidity (Blumberg et al., 2010), central line infections (Blumberg et al., 2005), transfusion reactions and red cell alloimmunization (Blumberg et al., 2003). We would suggest that prudence would dictate the use of leukoreduced whole blood and components in studies of resuscitation with transfusions. Except for the United States, universal leukoreduction has been implemented in almost all higher resource nations.

## Discussion

Animal model, *in vitro* and observational clinical studies demonstrate that infusion of ABO incompatible antigen and antibody increases bleeding, morbidity and mortality, interfering with hemostasis and host defenses. Patients of different ABO types have varied hemostatic function. The major limitation of these data is the lack of randomized trials, the reliance on observational data, and extrapolation from *in vitro* studies, no matter how high quality. Accounting for patient ABO type and quantity of infused ABO incompatible antigen and antibody will likely improve the likelihood of studies deriving true and clinically useful information for therapeutic strategies in hemostatic resuscitation. Future studies will be needed to assess the role of patient ABO blood group in bleeding, infection and thrombosis. Similarly, identification and quantitation of infusion of ABO mismatched antibody and cellular/soluble antigen will resolve the uncertainties concerning the importance of minimizing these potentially harmful substance that are transfused along with beneficial blood cells and pro-hemostatic proteins. Overwhelming benefits of leukoreduction in multiple clinical settings, demonstrated in many randomized trials in surgical patients, and high quality observational studies in all patients, would suggest that all transfusions to all patients should be leukoreduced, even in the trauma resuscitation setting.
